# Dietary microbes and functional dyspepsia: modulating the gut microecology for therapeutic benefit

**DOI:** 10.3389/fnut.2025.1625987

**Published:** 2025-08-19

**Authors:** Qi Huang, Fei You, Fengxia Liang, Chaoyang Ma

**Affiliations:** ^1^Department of Rehabilitation, The Central Hospital of Wuhan, Tongji Medical College, Huazhong University of Science and Technology, Wuhan, China; ^2^Department of Acupuncture and Moxibustion, Hubei University of Chinese Medicine, Wuhan, China

**Keywords:** gut microbiota, probiotics, prebiotics, dietary microbiota, functional dyspepsia

## Abstract

The pathogenesis of functional dyspepsia (FD) is closely associated with intestinal microecological alterations. Dietary microorganisms, capable of modulating gut microecology and thereby influencing gastrointestinal function, are being explored as a promising therapeutic strategy against FD. However, the precise mechanisms underlying how dietary microbes exert beneficial effects through microecological modulation, along with therapeutic protocols, remain incompletely defined. This article systematically reviews the manifestations of intestinal microecological imbalance in FD and its proposed pathogenic mechanisms. We critically examine the role of dietary microorganisms in mitigating FD through microecological regulation, addressing their potential mechanisms of action and clinical impacts. Integrating advances in emerging diagnostic technologies, we further discuss feasible approaches and potential targets for personalized FD management. Current controversies and challenges within this research domain are analyzed, alongside perspectives for translating these findings into clinical practice. Collectively, this review aims to provide a comprehensive theoretical framework and inspire insights for both in-depth research and improved therapeutic strategies for FD.

## 1 Introduction

Functional dyspepsia (FD) is a prevalent functional gastrointestinal disorder, it represents significant global health burden characterized by chronic upper abdominal pain, epigastric burning, postprandial fullness, or early satiation (1). Notably, approximately 80% of patients presenting with dyspeptic symptoms receive an FD diagnosis following exclusion of organic gastrointestinal pathology, with epidemiological studies reporting a general population prevalence approaching 16% (1). Emerging evidence has established a pivotal role of gut microbial dysbiosis in FD pathogenesis (2, 3). Mechanistic studies have revealed that gut microbiota disorders are not only associated with dysregulated immune responses and impaired mucosal barrier function in the gastrointestinal tract, but one of their central roles is also manifested in the interference with bidirectional communication in the gut-brain axis (4, 5). This interference is manifested by the dysbiotic flora through a variety of pathways such as altering the signaling of the enteric nervous system, affecting vagal tone, stimulating the release of inflammatory factors, and interfering with the metabolism of neurotransmitters, which ultimately leads to an abnormal transmission of information between the gut and the brain, amplifying the perception of nociception and affecting gastrointestinal dynamics regulation (4). In light of these findings, strategies based on modulation of the gut microbiota, particularly dietary microbial interventions such as probiotic supplementation, are increasingly becoming a promising therapeutic approach in FD clinics.

Gut microecology comprises a diverse ecosystem of microorganisms including bacteria, viruses, fungi, protozoa, and bacteriophages, with bacterial populations constituting over 90% of the total microbial community (6). Predominant bacterial phyla include *Firmicutes*, *Bacteroidetes*, *Proteobacteria*, *Actinobacteria*, with *Firmicutes* and *Bacteroidetes* representing the most abundant groups (6–8). These microbial communities exert profound effects on human physiology through nutrient metabolism, vitamin biosynthesis, immunomodulation, and maintenance of intestinal epithelial barrier integrity (9).

Dietary microorganisms exhibit modulatory effects on gut microbiota composition and ameliorate the symptoms of FD through multiple pathways, including competitive exclusion, production of bioactive metabolites, and immunomodulation (10). Researches have established that probiotic supplementation, such as *Lactobacillus gasseri LG21*, *Bacillus coagulans MY01* and *Bacillus subtilis MY02*, can restore microbial equilibrium, fortify intestinal barrier integrity, and enhance mucosal immunity—mechanisms that collectively mitigate intestinal inflammation and ameliorate FD-related symptoms (5, 11, 12). Consequently, targeted modulation of gut microbiota structure through dietary microorganisms represents a promising therapeutic approach. This strategy may offer novel intervention pathways for FD treatment.

Over the past 2 years, Tziatzios et al. and Shen et al. have systematically reviewed the mechanisms of action of probiotics in FD, respectively (13, 14). Tziatzios and colleagues conducted a comprehensive analysis of clinical trials investigating probiotic interventions for FD, concluding that current evidence remains insufficient to substantiate the clinical efficacy of probiotics in FD management (13). Concurrently, Shen’s team elucidated the mechanistic pathways through which probiotics may influence FD pathophysiology and symptom manifestation (14). Furthermore, Lacy et al. (15) have synthesized current therapeutic approaches for FD, including dietary modification, probiotic supplementation, antibiotic therapy, acid suppression, and neuromodulation. However, two key aspects remain unclear: the precise mechanisms by which dietary microorganisms modulate FD through gut microecological alterations, and the potential application of dietary microorganisms in FD diagnosis and treatment strategy.

This comprehensive review explores the impact and mechanisms of various dietary microorganisms on FD through their role in modulating the gut micro-ecological environment. It further investigates personalized treatment approaches and identifies potential therapeutic targets for FD, integrating advanced diagnostic technologies. We posit that systematic evaluation and clinical implementation of these multidisciplinary approaches will significantly enhance prognostic outcomes in FD management.

## 2 Epidemiologic characteristics of FD in association with intestinal microecology

### 2.1 Epidemiological characteristics of FD

FD exhibits a substantial global prevalence, with notable regional variations in its epidemiological profile. Among the general population, the overall prevalence of FD ranges from 10 to 20% (16). A multinational cross-sectional study conducted in the United States, Canada, and the United Kingdom revealed that the prevalence of FD meeting Rome IV diagnostic criteria averages approximately 10%, with country-specific rates of 12% in the United States, and 8% in both Canada and the United Kingdom (17). Clinically, FD manifests in distinct subtypes: postprandial distress syndrome (PDS), epigastric pain syndrome (EPS), and an overlapping subtype exhibiting features of both conditions. Epidemiological data indicate that PDS constitutes the predominant presentation, accounting for approximately 61% of cases, while EPS represents 18%, and the overlapping subtype comprises 21% of FD diagnoses. Evidence suggests these subtypes may demonstrate differential responses to therapeutic interventions, underscoring the importance of precise phenotyping in both clinical and research settings (17).

FD pathogenesis involves multiple etiological factors, notably psychological comorbidities, acute gastroenteritis, female sex, tobacco use, non-steroidal anti-inflammatory drug (NSAID) consumption, and *Helicobacter pylori* infection (1). Substantial clinical evidence demonstrates a significant association between *H. pylori* infection and FD symptom manifestation, with eradication therapy providing symptomatic relief in a subset of patients (18). Furthermore, psychiatric comorbidities including anxiety and depression constitute significant pathogenic contributors to FD. These psychological factors appear to modulate gastrointestinal sensory processing and motility through the gut-brain axis, ultimately influencing symptom perception and disease progression (19).

### 2.2 Association between FD and intestinal microecology in different populations

The relationship between FD and intestinal microecology exhibits significant heterogeneity across different populations. Age-related analyses reveal a progressive decline in beneficial intestinal bacteria among elderly populations, potentially elevating FD risk (20). This phenomenon may be attributed to age-associated physiological decline, including compromised intestinal barrier function, diminished microbial diversity, and impaired microbiota stability, collectively contributing to more pronounced FD symptoms and greater microecological dysbiosis in geriatric cohorts.

Gender-stratified analyses demonstrate a markedly higher FD prevalence in females compared to males, potentially mediated by hormonal fluctuations and psychological factors (1). Moreover, female FD patients tend to exhibit more significant intestinal microecological disturbances (1). Cross-ethnic and geographical comparisons indicate distinct patterns in FD prevalence and associated microbial characteristics. For instance, epidemiological data suggest stronger FD associations in Asian populations, while Western cohorts demonstrate differential microbiome-FD relationships, likely influenced by dietary patterns and lifestyle factors (18). Notably, patients with comorbid conditions such as diabetes mellitus and cardiovascular disease present with more complex FD pathogenesis. In these populations, metabolic dysregulation and pharmacological interventions may synergistically exacerbate FD risk while potentially confounding the interpretation of intestinal microecological imbalances (21, 22).

## 3 Pathogenesis of FD and interaction with intestinal microecology

The pathophysiological mechanisms underlying FD remain incompletely understood; emerging evidence suggests a predominant role of gut-brain axis dysregulation. The gut-brain axis constitutes a bidirectional neurohumoral communication network integrating neural, endocrine, and immune signaling pathways between the gastrointestinal tract and central nervous system (23). Current pathophysiological models implicate three principal components in FD pathogenesis: (1) psychological stress-mediated modulation of gut-brain signaling, (2) gut microbiota dysbiosis, and (3) immune system hyperreactivity (24, 25). These pathophysiological derangements manifest through several interrelated mechanisms: gastrointestinal motility abnormalities (particularly impaired gastric accommodation and delayed emptying), visceral hypersensitivity (with heightened duodenal sensitivity to acidic and lipid stimuli), intestinal barrier dysfunction, and low-grade mucosal inflammation (26–28). Crucially, the sustained low-grade inflammatory state and compromised epithelial barrier integrity collectively contribute to aberrant afferent signaling through both neural and humoral pathways, ultimately generating the characteristic symptom constellation of FD (29, 30).

### 3.1 Mechanisms of interaction between FD and intestinal microecology

An imbalance in gut microbiota may be one of the significant contributing factors to the development of FD. Comparative analyses reveal significant compositional differences between the gut microbiota of FD patients and healthy controls (31–33). These taxonomic alterations may contribute to disease progression through multifactorial mechanisms, including compromised intestinal barrier integrity, immune dysregulation, and disrupted neuro-endocrine signaling pathways (31, 34). Microbial profiling reveals significantly increased diversity in FD patients relative to healthy individuals (35). Notably, the microbiota of FD patients demonstrates marked reductions in beneficial bacterial genera (e.g., *Bifidobacterium* and *Lactobacillus* spp.), coupled with elevated levels of potentially pathogenic microorganisms (e.g., *Escherichia coli* and *Enterococcus* spp.) (36). Furthermore, metagenomic analyses have identified significant perturbations in the *Firmicutes*/*Bacteroidetes* ratio, a recognized microbial community stability index (37). The resultant microbial imbalance promotes the secretion of enterotoxins (including SEA/SEB), potentially inducing duodenal mucosal barrier dysfunction and localized immune-inflammatory responses (38). Additionally, microbial metabolites may directly stimulate intestinal afferent nerve terminals, contributing to visceral hypersensitivity and gastrointestinal motility abnormalities (19). An imbalanced gut microbiota can affect the production and metabolism of neurotransmitters such as serotonin, dopamine, etc. This can indirectly activate the vagus nerve and enteric nervous system, thereby altering signal transmission with the central nervous system, leading to symptoms like early satiety, postprandial bloating, anxiety, and depression in FD patients (38, 39).

**Figure 1:**
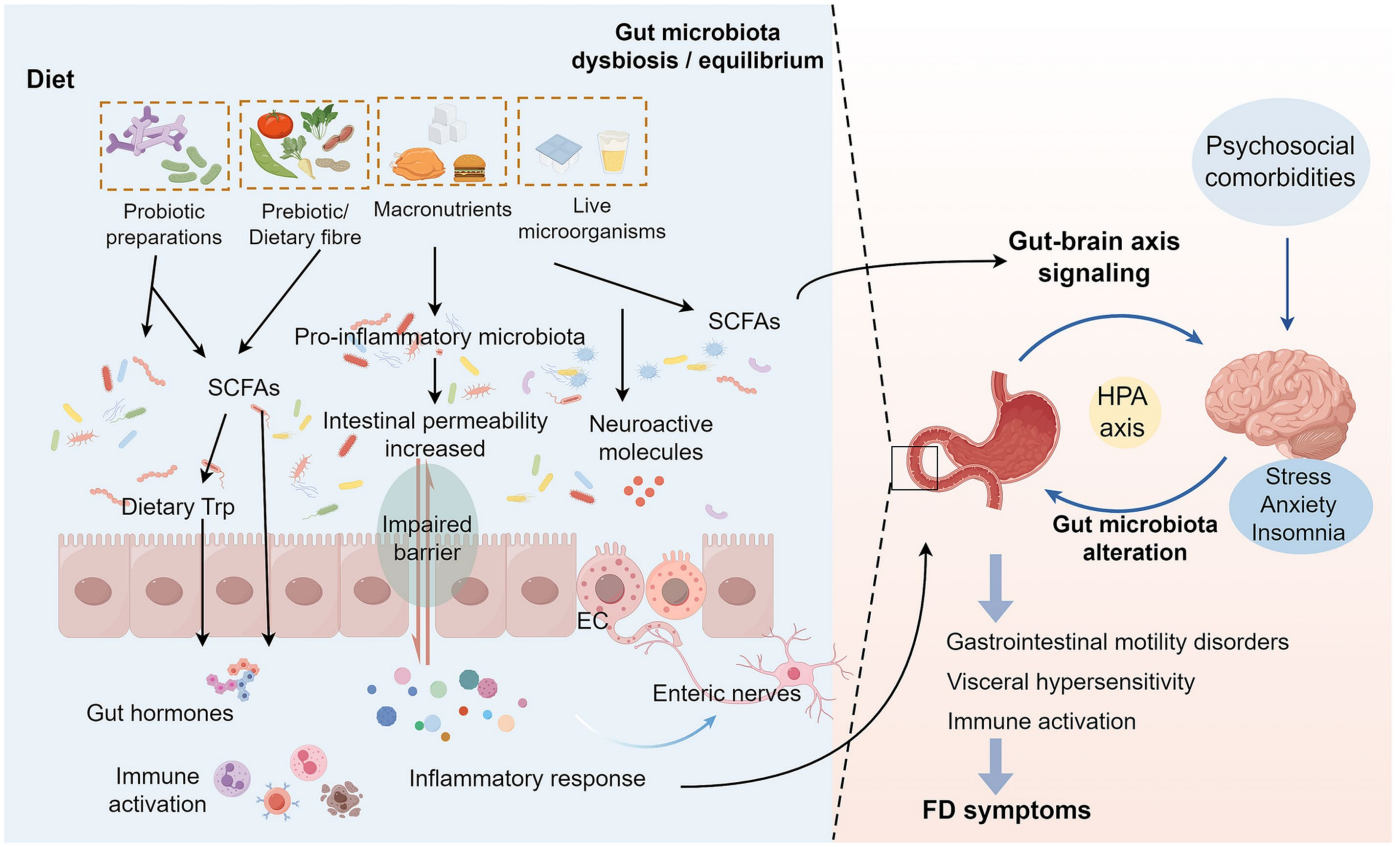
Interaction mechanisms between FD and intestinal microecology. Changes in the dietary structure of FD patients lead to a decrease in beneficial intestinal bacteria and an increase in pro-inflammatory bacteria, causing changes in SCFAs and other metabolites of the intestinal flora. On the one hand, these changes will affect the levels of various intestinal bioactive factors, resulting in impaired intestinal barrier function and increased permeability, which in turn activate the local inflammatory signaling pathway in the intestinal tract, allowing inflammatory factors and bacterial products to enter the blood circulation, and ultimately triggering systemic immune-inflammatory response and metabolic abnormalities, leading to abdominal pain, early satiety and other core symptoms of FD. On the other hand, an imbalanced intestinal microecology will interfere with neurotransmitter release, affecting the HPA axis function by stimulating the vagus nerve and enteric nervous system, leading to abnormal signaling interactions between the gut and the brain, which not only amplifies pain perception, but also disrupts gastrointestinal dynamics regulation (24). In addition, somatization symptoms such as anxiety and depression accompanying FD patients will also activate the HPA axis, further exacerbating intestinal flora dysbiosis and destroying the intestinal barrier, thus forming a vicious circle of mutual causation (19). Dietary microorganisms maintain intestinal homeostasis by regulating the balance of intestinal microecology and the production of SCFAs, repairing the intestinal barrier function, and regulating the immune response and metabolic status of the host (64, 70). FD, Functional dyspepsia; SCFAs, Short-chain fatty acids; EC, Enteroendocrine cells; HPA axis, Hypothalamic–Pituitary–Adrenal axis.

On the other hand, alterations in the intestinal microenvironment of FD patients, including disturbances in gastric acid secretion and gastrointestinal motility, may significantly impact microbial survival and proliferation, thereby exacerbating dysbiosis. Mounting evidence indicates that delayed gastric emptying promotes prolonged food retention within the gastrointestinal tract, creating favorable conditions for bacterial overgrowth and subsequent microbial community structural modifications (26, 40). Furthermore, FD patients frequently exhibit anxiety, depression, and somatic symptoms. This activates the hypothalamic–pituitary–adrenal (HPA) axis and sympathetic nervous system (SNS), resulting in sustained elevation of cortisol levels. Increased cortisol suppresses intestinal tight junction proteins and activates the NF-kB inflammatory signaling pathway, thereby compromising the intestinal barrier (19, 41). Notably, stress reduces populations of beneficial bacteria (e.g., *Lactobacillus*, *Bifidobacterium*) while increasing pro-inflammatory bacteria (e.g., *Proteobacteria*) in FD patients (19). This shift alters gut-derived metabolites (including short-chain fatty acids, SCFAs, and gamma-aminobutyric acid, GABA), which in turn stimulates the vagus nerve to modulate the HPA axis, exacerbating core FD symptoms (41). Moreover, intestinal barrier impairment increases permeability, allowing bacterial products to enter systemic circulation. This activates body-wide immunoinflammatory and metabolic responses, exerting broader pathophysiological effects in FD (42). Future research should prioritize mechanistic studies to elucidate precise microbiota-host crosstalk in FD pathogenesis (Figure 1).

### 3.2 Intestinal microecological imbalance in FD

Intestinal microecological imbalance, via alterations in specific bacterial community structures and disruption of metabolic products, directly mediates the core symptomatic manifestations of FD. Changes in the gut microbiota correlate with the severity of FD symptoms (43). Research confirms that over-proliferation of Streptococcus in the duodenum exhibits a significantly positive correlation with postprandial fullness and early satiety (44). Its mechanism is linked to bacterial-mediated degradation of tight junction proteins, resulting in disruption of the intestinal mucosal barrier and activation of local immune-inflammatory responses, ultimately triggering epigastric burning pain and postprandial bloating (44). Concurrently, depletion of butyrate-producing bacteria (*Butyricicoccus*, *Prevotella*) leads to insufficient synthesis of SCFAs, impairing the repair capacity of intestinal epithelial cells and further exacerbating abdominal distension and discomfort (45). SCFAs serve not only as the primary energy source for intestinal epithelial cells but also play a crucial role in regulating intestinal barrier function and immune responses (46, 47).

Notably, reduced abundance of *Veillonella* is closely associated with delayed gastric emptying. Dysfunction in the metabolism of its products, such as hydrogen sulfide, can directly impact gastrointestinal motility, manifesting as food retention and nausea (44). Furthermore, translocation of oral-derived Neisseria to the duodenum exacerbates symptoms of PDS, specifically early satiety and epigastric pain, by inducing mucosal stress through enhanced protease activity (45). Simultaneously, fecal *Butyricicoccus* levels show a strong negative correlation with symptom severity, underscoring the driving role of microbial metabolic imbalance in persistent symptoms (45). Clinical studies further reveal that FD patients exhibit reduced concentrations of bile salts within the intestinal environment during fasting, coupled with increased expression of the vitamin D receptor (48). This impacts fat digestion and the secretion of intestinal hormones, further worsening dyspeptic symptoms in FD patients (48).

Collectively, the above evidence demonstrates that intestinal microecological imbalance can regulate the symptomatic expression of FD through multiple dimensions. This mechanism relates to intestinal microecological imbalance causing damage to the intestinal mucosal barrier, which then triggers chronic micro-inflammation and immune dysfunction (10, 49).

## 4 Dietary microbial modulation in the treatment of FD

Dietary microorganisms demonstrate considerable diversity and can be systematically classified into four primary categories: probiotics, prebiotics, live microorganisms, and macronutrient-associated microorganisms.

(1) Probiotics encompass multiple bacterial genera, including *Lactobacillus*, *Bifidobacterium*, and *Bacillus*. Specifically, lactic acid bacteria (e.g., *Lactobacillus acidophilus* and *Lactobacillus plantarum*) acidify the intestinal lumen through organic acid (particularly lactic acid) production, thereby inhibiting pathogenic bacterial growth while concurrently modulating intestinal immune responses (14). *Bifidobacterium bifidum* enhances intestinal barrier integrity, facilitates nutrient absorption, and maintains microbial homeostasis through immunomodulatory interactions (37). *Bacillus* spp. spores exhibit intestinal germination capacity, subsequently influencing microbiota composition and host immunity (50).

(2) Prebiotics represent selectively fermentable substrates (e.g., inulin, oligofructose) that preferentially stimulate growth of beneficial microbiota (particularly *Bifidobacterium* and *Lactobacillus* spp.), thereby indirectly modulating intestinal ecology (51).

(3) Live microorganisms predominantly present in fermented foods competitively exclude enteropathogens while stimulating SCFAs production and reinforcing intestinal barrier function (52).

(4) Macronutrients demonstrate significant microbiota-modulating effects: Carbohydrates (e.g., starch, glucose, fructose, lactose) undergo colonic microbial fermentation, serving as primary energy substrates for gut microbiota (53). Proteinaceous substrates elevate SCFAs concentrations while enhancing barrier function and immunological regulation (54–56). Dietary lipids exhibit differential effects: saturated fats promote *Bilophila* and *Enterobacteriaceae* proliferation with concomitant pro-inflammatory effects, whereas omega-3 polyunsaturated fatty acids (PUFAs) increase *Bifidobacterium* and *Akkermansia* abundance while attenuating inflammation and improving barrier integrity (57). These classifications and corresponding microecological impacts are systematically summarized in Table 1.

**Table 1:** Classification of dietary microorganisms and their role in intestinal functions.

Microbial species	Genus/species	Typical strains	Functional role in intestinal
Probiotics	*Lactobacillus rhamnosus*	LGG	Enhance intestinal barrier function (129, 130)
	*Lactobacillus plantarum*	*L. plantarum* L15; LR	Inhibit LPS-mediated NF-κB activation and improve intestinal flora dysbiosis (131, 132)
	*Bifidobacterium lactis*	*B. lactis* A6	Regulate immune activation and increase SCFA production (133)
	*Bifidobacterium longum*	*B. longum* BB536	Improve intestinal permeability and inhibit intestinal barrier damage (134)
	*Lactobacillus casei*	Lcr35	Reduce inflammation scores and restore bacterial homeostasis (135)
	*Lactobacillus delbrueckii*	pExu:hsp65	Reduce inflammatory infiltration and increase intestinal IgA levels (136)
	*Bifidobacterium infantis*	JYBR-190	Protect intestinal mucosa from pathogen damage and enhance antimicrobial activity (137)
	*Lactobacillus acidophilus*	NCFM	Improve metabolic disorders (e.g., type 2 diabetes mellitus) and enhance host metabolic regulation (138, 139)
Prebiotic-related microorganisms	*Bifidobacterium* spp. *Bacteroides* spp.	*B. adolescentis* *B. breve* *B. xylanisolvens*	Selectively utilizes FOS/GOS and promotes SCFA production, breaks down dietary fiber, and maintains intestinal homeostasis (140–143)
Live microorganisms (fermented foods)	*Lactococcus* spp. *Weissella cibaria*	*L. lactis* *L. mesenteroides*	Enhances host immunomodulation (144–146)
Macronutrient-related microorganisms	*Prevotella* spp. *Clostridium* spp. *Ruminococcus* spp.	Tryptophan	Catabolizes complex carbohydrates (e.g., cellulose, resistant starch) and regulates intestinal energy balance (147–149)
Others	*Enterococcus faecium*	C171	Competitively inhibit pathogen colonization and modulate host immune response (150, 151)

SCFA, Short-chain fatty acid; LPS, Lipopolysaccharides; NF-κB, Nuclear Factor kappa-B; FOS, Fructooligosaccharide; GOS, Galactooligosaccharides; LGG, *Lactobacillus rhamnosus* GG; *L. plantarum* L15, *Lactobacillus plantarum* L15; LR, *Lactobacillus plantarum* LR002; *B. lactis* A6, Bifidobacterium subsp. lactis A6; *B. longum* BB536, *Bifidobacterium longum* BB536; Lcr35, *Lactobacillus casei* variety rhamnosus; pExu:hsp65, *Lactobacillus delbrueckii* CIDCA 133; JYBR-190, *Bifidobacterium lactis* JYBR-190; NCFM, *Lactobacillus acidophilus* NCFM; C171, *Enterococcus faecium* C171.

### 4.1 Influence of dietary microorganisms on gut microecology and its health implications

Dietary microorganisms and their metabolic activities play a pivotal role in shaping gut microecology and influencing overall host health. This influence begins with the microbial community itself: specific dietary components (such as fiber and polyphenols) can significantly regulate the composition and functional activity of the gut microbiota. For instance, they promote the growth of beneficial bacteria (e.g., *Bifidobacteria* and *Lactobacilli*), suppress the proliferation of pathogens, and stimulate physiological processes like brown fat activation (58–60). Certain *Lactobacilli* can directly enhance intestinal barrier function, defending against pathogens and toxins (61, 62). The resulting changes in microbial structure profoundly affect key outputs of the gut microenvironment—particularly the levels and types of fermentation products such as SCFAs (63, 64). These dietary fiber-derived SCFAs (e.g., butyric acid, acetic acid, propionic acid) not only serve as an important source of energy for colonocytes and maintain a healthy intestinal pH (65, 66), but also act as important signaling molecules. They regulate host immune responses and intestinal epithelial integrity through G protein-coupled receptors (GPCRs) (63, 64). Furthermore, microbially derived secondary bile acids inhibit specific pathogens (e.g., *Clostridia*) and regulate lipid metabolism by activating the FXR receptor (67). The gut microbiota also influences host nutritional status through the synthesis of essential vitamins (e.g., vitamin K, folate, biotin, riboflavin). Conversely, imbalances (such as vitamin A deficiency) can feedback to affect microbial abundance (e.g., increased *Bacteroides fragilis*) and bile acid metabolism (68, 69).

The impact of dietary microbial metabolites extends far beyond the local environment of the gut. They can cross the intestinal barrier into the body circulation and systematically modulate the immune response and metabolic status of the host (64, 70). For example, dietary amino acid-derived metabolites can interact with various pattern recognition receptors [including Toll-like receptors, autoinducer-2 (AI-2), and NOD-like receptors (NLRs)] and activate signaling pathways, such as the aromatic hydrocarbon receptor (AhR) and serotonin/5-hydroxytryptophan (5-HT), which directly or indirectly shape the intestinal mucosal immunity and bacterial homeostasis, and jointly maintain intestinal homeostasis (70). Further, these microbial metabolic signals also form the basis of gut-brain axis communication. Substances produced by gut flora act on the central nervous system through neural, immune, and endocrine pathways, influencing brain function, behavior, and even mood regulation, and potentially playing a role in neurodevelopment and the pathology of certain neuropsychiatric disorders (71, 72).

**Figure 2:**
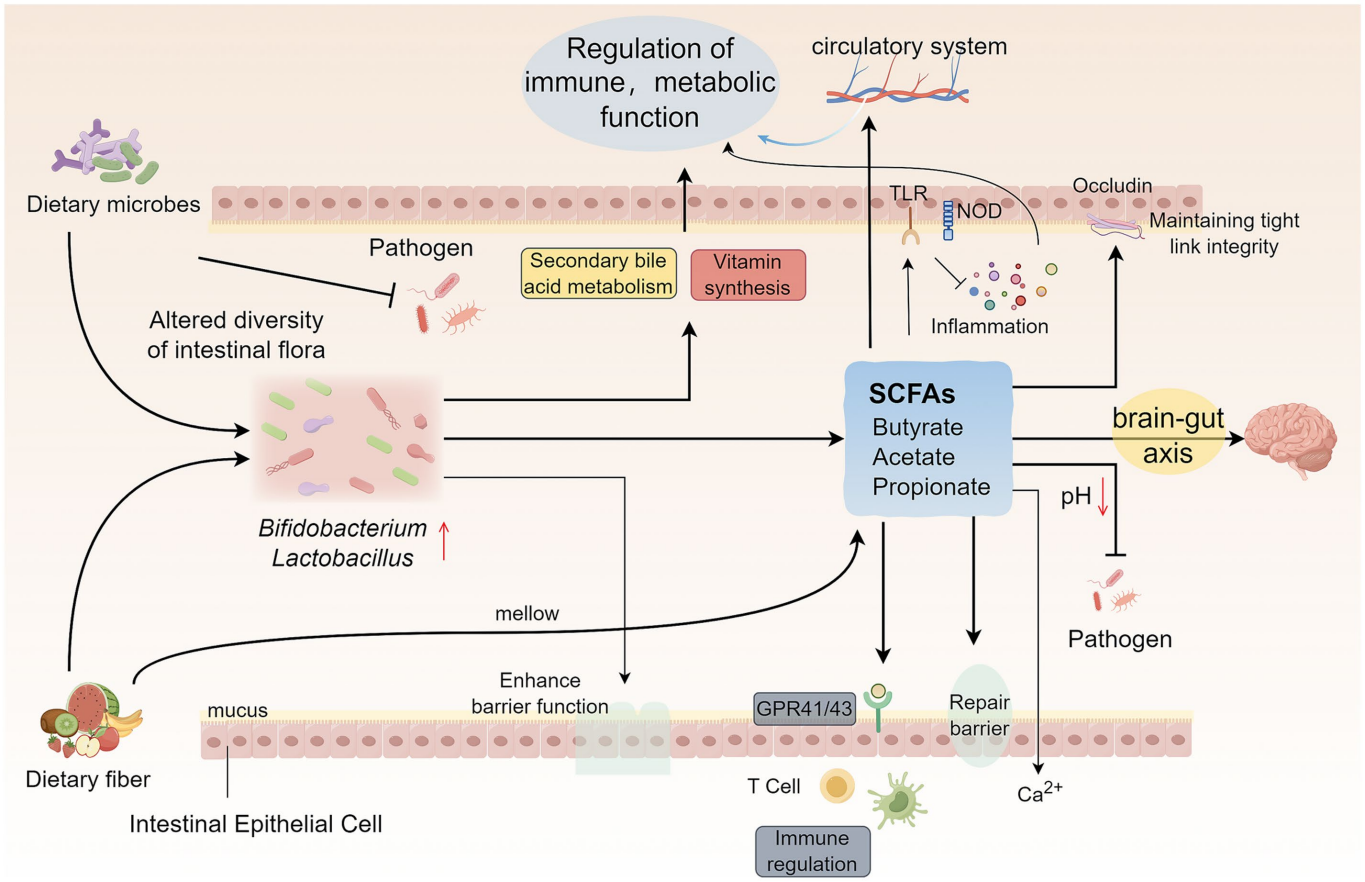
Mechanistic insights into dietary microorganism-mediated modulation of intestinal microecology. Dietary microorganisms influence intestinal microecology in four main ways: (1) Dietary fiber and polyphenols promote the proliferation of beneficial bacteria (e.g., *Bifidobacterium* and *Lactobacillus*) and inhibit pathogenic bacteria by regulating the composition of the intestinal flora, and at the same time enhance the intestinal barrier function, promote the metabolism of secondary bile acids, and assist in the synthesis of a variety of vitamins. (2) Fermented dietary fiber produces metabolites, such as SCFAs, to maintain the intestinal environment in a homeostatic state, increase the integrity of intestinal tightly linked proteins, promote the recovery of intestinal barrier function, and influence systemic immune regulation through GPCRs. (3) SCFAs exert both direct and indirect immunomodulatory effects on intestinal mucosal immunity and inflammatory responses through their interactions with host pattern recognition receptors, including TLRs and NLRs. Following their absorption, these microbial metabolites enter the systemic circulation, thereby influencing systemic immune responses and metabolic status. (4) Dietary microorganisms and metabolites such as SCFAs interact through the gut-brain axis to influence brain function with the help of the neuro-immune-endocrine network. SCFAs, Short-chain fatty acids; TLRs, Toll-like receptors; NLRs, NOD-like receptors; GPCRs, G protein-coupled receptors.

Thus, dietary microbes profoundly influence the intestinal microecological environment and ultimately have a broad and far-reaching impact on host metabolic health, immune defense, and even neurological function through multilevel fine-tuned mechanisms including shaping colony structure, producing key metabolites, and mediating complex gut- neurological interactions (Figure 2). A deeper understanding of these interactions provides a key scientific basis for the development of diet-based intervention strategies to promote human health.

### 4.2 Efficacy of dietary microbial interventions for FD

Dietary microorganisms have shown potential in the treatment of FD, with mechanisms of action involving repair of the intestinal mucosal barrier (e.g., probiotics improve permeability and protect the mucosa through enhancement of intercellular junctional proteins) (73), immunomodulation (e.g., modulation of immune cell activity and cytokine secretion to attenuate inflammatory responses) (14), and synergization of the neuroendocrine system (e.g., influencing gastrointestinal hormone secretion, kinetic-sensory function to alleviate symptoms) (37). As representatives of beneficial active microorganisms, specific probiotic strains (e.g., *Bifidobacterium*, *Lactobacillus acidophilus*, Probiotics *Bacillus*) have been widely demonstrated to alleviate the symptoms of FD due to their ability to regulate the microecological balance of the intestinal tract (3, 12, 74). For example, clinical studies have shown that a combination preparation containing *Bacillus coagulans MY01* and *Bacillus subtilis MY02* significantly improved abdominal pain and fullness in patients, and the mechanism may involve its regulation of Th17 immune signaling-mediated flora modulation (12); whereas a probiotic combination containing four strains of *Lactobacillus rhamnosus LR04*, etc. was effective in reducing symptoms such as bloating and nausea, especially in patients with the PDS subtype associated with gastrointestinal dynamics sensitivity, and its mechanism of action may be closely related to the regulation of flora composition and reduction of visceral hypersensitivity (74). In addition, *Lactobacillus reuteri DSM 17938* strain has shown specific effects on infantile abdominal pain (75, 76). As a complement to the probiotic strategy, the prebiotic kelp polysaccharide (*Laminarin*) corrects FD-associated flora imbalances (e.g., an imbalance in the ratio of *phylum Anabaena* to *phylum Thicket*) and ameliorates symptoms by modulating corticosterone hormone levels and inhibiting 5-HT3 receptor overexpression (37). Equally noteworthy is dietary intervention; although the low-FODMAP (fermentable oligo-, di-, mono-saccharides and polyols) diet has been less studied in FD than in irritable bowel syndrome (77, 78), this strategy has also been shown to be effective in relieving symptoms such as bloating and abdominal pain in FD patients by reducing intestinal gas production and optimizing gastrointestinal function (79, 80). It should be noted that the efficacy of existing studies on probiotics, prebiotics, and specific dietary patterns varies somewhat, which may be influenced by a variety of factors such as strain selection, dosage, treatment duration, and individual patient differences.

### 4.3 Long-term impact of dietary microbial regulation on intestinal microecology

The long-term impact of dietary microbial modulation on the intestinal microecology of patients with FD centers on three aspects: repair of the mucosal barrier, remodeling of the flora structure and function, and modulation of the neuroimmunity. Significant microecological imbalances are present in patients with FD, including barrier disruption due to aberrant expression of duodenal tight junction proteins, and dysregulation of the gastric and duodenal bacterial flora (e.g., increased *Streptococcus* and decreased *Prevotella*), >60% concomitant small intestinal bacterial overgrowth, and diminished anti-inflammatory and barrier repair functions due to inadequate synthesis of SCFAs (10, 73, 81, 82). Long-term probiotic interventions (e.g., supplementation with *LG21* strains over 8–12 weeks) ameliorate this imbalance through multiple mechanisms. First, for barrier repair, up-regulation of tight junction proteins (ZO-1, Claudin-8) reduces intestinal permeability and decreases antigen penetration (11, 81–83). Second, for flora optimization, the abundance of Lactobacillus, Bifidobacterium, and SCFA-producing bacteria (e.g., *Ruminococcaceae*, *Prevotella*) was elevated by inhibiting pathogenic bacterial colonization, and the intestinal epithelium was supplied with energy by increasing SCFAs such as butyric acid (51, 84, 85). Third, neuroimmunomodulation promotes anti-inflammatory factors and modulates the brain-gut axis via neurotransmitters (GABA, serotonin) to alleviate visceral hypersensitivity (82). In addition, studies have shown that specific strains such as *L. gasseri OLL2716* significantly reduced bile reflux and repaired the mucosa in non-*H. pylori*-infected FD patients, with a symptom elimination rate of 35.3% in the 12-week intervention (17.3% in the placebo group) (11), while multi-strain combinations (e.g., *Lactobacillus*+*Bifidobacterium*) resulted in >70% relief of abdominal distension and abdominal pain through synergistic potentiation (74, 84, 86).

Long-term stability and key influences of the intervention need to be focused on: continued supplementation for >6 months maintains colony alpha diversity and abundance of SCFA producers (*Prevotella*, etc.), and enhances colony structure against fluctuations (85, 87). However, host age (older adults cause resistance to colonization due to increased *Bacteroidetes*) (88, 89), medication history (antibiotics/PPIs diminish effectiveness) (90), and dietary patterns (response rates are lower in those high in animal protein than in those high in fiber) (91) significantly influence individual efficacy. Nevertheless, excessive or inappropriate microbial interventions may potentially disrupt microbial equilibrium or promote antimicrobial resistance, necessitating careful protocol design and longitudinal monitoring of therapeutic outcomes.

## 5 Gut microecology diagnostic techniques in FD

### 5.1 Gut microecological assessment to assist in the diagnosis of FD

Gut microecological assessment has important potential in the diagnosis of FD. With the help of 16S rRNA sequencing, macrogenomics and metabolomics, the composition, diversity, functional activity and changes in their metabolite profiles of the intestinal microbiota can be systematically analyzed to gain insights into the development of FD (35, 92). Specifically, 16S rRNA sequencing is used to analyze the species and abundance of the flora, macrogenomics reveals the gene functions and metabolic pathways of microorganisms, and metabolomics monitors the dynamic changes of microbial metabolites (35, 92). The application of these techniques has clearly revealed that the duodenal microbiota of FD patients at the genus level is characterized by a specific flora structure and associated microbial functions (35), and these findings provide new perspectives for understanding the pathogenesis of FD. In addition, indicators of intestinal barrier function (e.g., serum connexin, fecal calreticulin) can indirectly reflect the health status of the intestinal microecology and provide valuable additional information for FD diagnosis (73). Current studies consistently show that intestinal microecological imbalance in FD patients often coexists with impaired intestinal barrier function. Of interest, restoring microbial balance through interventions such as probiotics and prebiotics has been shown to promote the production of SCFAs, improve the diversity and stability of the bacterial flora, and ultimately enhance intestinal barrier function (93, 94).

### 5.2 Novel gut microbial detection technologies in FD

Recent advances in gut microbial detection technologies have significantly expanded research and diagnostic capabilities for FD. Emerging artificial intelligence (AI)-based analytical approaches enable rapid and precise evaluation of extensive gut microbiome datasets, facilitating identification of specific microbial signatures associated with FD pathogenesis. Application of AI algorithms to comparative analyses of gut microbiota from FD patients versus healthy controls has revealed distinct microbial consortium patterns that correlate strongly with disease progression. These microbial biomarkers show potential for early diagnostic applications and longitudinal disease monitoring (95).

High-resolution microendoscopy (HRME) represents an innovative imaging modality that provides real-time, cellular-level visualization of intestinal mucosa. This technique enables detection of subtle architectural alterations in mucosal microstructure resulting from gut microbial dysbiosis. In FD investigations, HRME has demonstrated significant abnormalities in duodenal mucosal epithelial cell morphology and spatial organization, which may reflect inflammatory processes and compromised barrier function secondary to microbial imbalance (96). Endomicroscopic analyses have further revealed a higher prevalence of gastric metaplasia in the duodenal bulb among FD patients compared to asymptomatic controls (97), suggesting potential pathophysiological relevance and future therapeutic targeting.

In addition, Spectroscopic methodologies, including elastic scattering spectroscopy and angle-resolved low-coherence interferometry, offer additional diagnostic precision by characterizing tissue optical properties to evaluate mucosal microstructure and biochemical composition. These non-invasive techniques enhance detection of early tissue-level changes induced by microbial dysregulation, providing valuable complementary data for FD diagnosis (98).

### 5.3 Biomarkers of FD and intestinal microecology

The identification of FD-related intestinal microecological biomarkers is significant in enabling early diagnosis, monitoring the condition and guiding treatment. Current research has identified several promising candidate biomarkers. At the microbial level, decreased abundance of specific gut bacteria (e.g., *Bifidobacterium bifidum*, *Lactobacillus lactis*) as well as an increase in harmful bacteria, such as *Escherichia coli*, are associated with the pathogenesis of FD, and the pattern of their imbalance can serve as an important indicator of microecological homeostasis (36, 45). Among the microbial metabolites, SCFAs, especially the decreased levels of butyric acid due to the reduction of butyric acid-producing bacteria, are of great interest (99). Decreased levels of SCFAs have been shown to affect intestinal barrier function and immune regulation, and have been associated with the symptomatic manifestations of FD (66). In addition, indicators reflecting local and systemic immune activation status in the gut have potential. For example, FD patients are seen to have increased infiltration of immune cells such as mast cells and eosinophils in the intestinal mucosa, and elevated serum levels of pro-inflammatory cytokines (e.g., interleukin-6, tumor necrosis factor-α) (5, 73). Meanwhile, the low-grade inflammation at the duodenal site and the abnormal alteration of tight junction protein expression further corroborate the central role of immune dysregulation of the intestinal microenvironment in the pathophysiologic process of FD (100).

Currently, most candidate biomarkers in the field of FD are still in the exploratory phase of research. To advance their clinical use, future rigorous work is needed for validation: this includes assessing their diagnostic sensitivity and specificity in large, independent and representative cohorts of FD patients and control populations. Further long-term follow-up studies are needed to clarify whether dynamic changes in these markers are associated with fluctuations in FD symptoms, disease progression or remission, and the efficacy of different interventions (e.g., probiotics, prebiotics, or dietary modifications). To overcome the limitations of single-marker performance, improve overall accuracy and lay the foundation for precision medicine, it is necessary to integrate multidimensional data from the microbiome, metabolome (e.g., SCFAs, etc.), immunome, and host genome, and to combine them with exhaustive clinical phenotypic and lifestyle information to construct diagnostic, typing, or prognostic models by using advanced methods such as machine learning. These integrated approaches are expected to yield reliable performance data and enable clinical translation, enhancing predictive power through complementary information. However, the high financial and time costs involved in these ambitious research programs, as well as the high complexity of integrating, processing, and analyzing data from multiple sources, pose potentially significant obstacles.

### 5.4 Personalized dietary microbial regulation programs for patients with FD

The management of patients with FD is increasingly focused on precision nutritional interventions based on the gut microbiota. Given the complex interactions between gut microbes and their hosts, as well as the significant individual differences in microecological profiles, symptom manifestations, and therapeutic responses (101, 102), the design of personalized dietary microbial modulation protocols is key. The basis of this lies in the accurate assessment of the patient’s intestinal microecology (e.g., 16S rRNA sequencing and macro-genomics to reveal the imbalance in flora composition, diversity, and function), and the in-depth integration of individualized factors such as symptomatic characteristics, dietary patterns, lifestyle habits, and comorbidities (36). For example, for specific flora imbalances (e.g., *Bifidobacteria* deficiency), the corresponding probiotic preparation can be supplemented (13). Specific to symptoms, patients with dyskinesia who are predominantly postprandial fullness and early satiety may use probiotics that improve gut motility, like *Lactobacillus rhamnosus GG* (*LGG*) which promotes mucin production by modulating the 5-HT4 receptor and flora to alleviate constipation (103), or probiotic combinations (*Lacticaseibacillus paracasei JY062* and *Lactobacillus gasseri JM1*) restores motility by bi-directional modulation of pro-motility factors (gastric motility, gastrin, 5-hydroxytryptamine) and inhibitory factors (peptide YY, nitric oxide) (104). At the same time, the choice of intervention modalities needs to take into account dietary interactions with flora and hormones, e.g., low FODMAP diets and specific dietary patterns (e.g., polyphenol-rich anti-inflammatory diets) can attenuate associated symptoms or inflammation by modulating flora and immune responses (60, 105). For special groups (e.g., FD patients with comorbid diabetes), additional assessment of the impact of interventions on glycemia is required.

In order to consolidate efficacy and reduce the risk of relapse, personalized regimens need to focus on their long-term effects and be clinically stratified: e.g., specific single strains (e.g., *L. plantarum*) are preferred for the PDS type, whereas prokinetic drugs may be used in combination with the predominantly EPS type (74). The use of enteric capsule technology is essential to ensure effective colonization of key areas of the probiotic (e.g., duodenum) (106). The use of a synbiotic strategy (probiotics in combination with specific prebiotics such as inulin) significantly increases the production of SCFAs and significantly reduces relapse rates (107). Long-lasting colonization techniques and customized strain combinations based on host gut type need to be developed in the future to enhance the efficacy of the intervention (91, 108). In clinical practice, a phased intervention is recommended: an initial phase with a high-dose multi-strain combination (e.g., *Lactobacillus gasseri* + *Bifidobacterium lactis*) for rapid symptomatic improvement, and a maintenance phase with a low-FODMAP diet and prebiotics to stabilize the flora (74, 86). This precise protocol, which combines assessment, individualized analysis of intervention elements, and multi-level implementation strategies, is expected to significantly optimize the overall treatment outcome of FD and improve patients’ quality of life (4, 109, 110).

In addition, fecal transplantation (FMT) technology has also demonstrated potential for the treatment of FD by correcting host intestinal dysbiosis. The core technology of FMT, in particular optimized washout microbiota transplantation (WMT), involves the introduction of functioning microbial communities into the patient through the upper gastrointestinal tract (e.g., naso-duodenal tube) or lower gastrointestinal tract (e.g., colonoscopy) routes by means of a rigorously screened, healthy donor fecal flora, aiming at reestablishing a healthy microecological balance in the patient. The aim is to re-establish a healthy micro-ecological balance. Clinical studies have shown that more than 62.5% of FD patients experience symptomatic improvement after three FMTs, with a significant improvement in quality of life (111). Randomized controlled trials further confirmed that the symptom disappearance rate and total relief rate of patients in the WMT group were significantly better than those in the conventional probiotic group (112). This may be related to the increased abundance of *Lactobacillus* and *Bacteroides* and decreased *Streptococcus* spp. in patients after FMT, as well as increased levels of metabolites related to digestion and mucosal repair (e.g., arginine, N-acetylglutamic acid) (111). The short-term effects of FMT are clear and the safety is relatively controllable, but the sample sizes of the studies are small, the long-term efficacy data are insufficient, and individualized regimens are missing. Addressing the maintenance of long-term efficacy, exploring individualized protocols, and improving the convenience of the technology are key to promoting its clinical application.

## 6 Points of controversy and future prospects

### 6.1 Controversies and challenges of dietary microbial modulation in FD

The application of dietary microbial modulation in FD presents substantial controversies and unresolved challenges. First, while certain studies demonstrate symptom amelioration through probiotic interventions, considerable heterogeneity exists among clinical outcomes, with multiple trials failing to establish statistically significant efficacy (113). This variability likely stems from confounding factors including strain selection, dosage regimens, treatment duration, and interindividual differences in microbiome composition. Particularly, baseline microbiota profiles and comorbid conditions may further influence therapeutic response (64). Consequently, precision microbiota-based interventions necessitate comprehensive characterization of individual microbial ecosystems (114).

Second, FD represents a complex gut-brain axis disorder with incompletely elucidated pathophysiological mechanisms, resulting in the absence of consensus regarding optimal microbial modulation strategies (115). Critical knowledge gaps persist concerning selection criteria for specific microbial taxa, optimal combinatorial approaches, and evidence-based dosing protocols. Furthermore, longitudinal safety assessments remain imperative, particularly regarding potential iatrogenic dysbiosis and antimicrobial resistance development (4, 80, 116). Of particular clinical relevance is the established role of psychological factors in FD pathogenesis; however, mechanistic understanding of microbiota-psychology crosstalk remains limited, presenting a fundamental barrier to developing integrated biopsychosocial treatment paradigms (19).

### 6.2 Future directions and technological breakthroughs in intestinal microecology research

In the future, intestinal microecology research will develop in the direction of deeper and more precise. In terms of technology, the integration of multi-omics technologies will become an important trend. By combining multiple technologies such as macrogenomics, transcriptomics, proteomics and metabolomics, the multilevel and dynamic interaction network between gut microbes and hosts will be systematically analyzed. This integrated analysis will profoundly reveal the functional mapping of gut microecology and its mechanism of action in health and disease states (117–119). Critically, the research will focus on the complex interactions between the gut microbiota and the host immune and neuro-endocrine systems. The “complexity” of this interplay is reflected in the fact that, on the one hand, the gut flora acts as a key signaling molecule through its metabolites (e.g., short-chain fatty acids, tryptophan, neurotransmitter precursors, etc.), and at the same time, acts on the immune cells of intestinal mucosa, cells of the enteric nervous system, and cells of the enteroendocrine system, which together maintain the function of intestinal barriers, regulate the level of local and systemic inflammation levels, influence neural signaling (e.g., via vagal pathways) and secretion of key hormones (e.g., cortisol) (38, 70, 120, 121).

On the other hand, host status (e.g., dietary structure, stress level, etc.) profoundly affects the gut microenvironment and neuroendocrine activity, which in turn regulates the composition and function of the microbiota; at the same time, cytokines produced by the host immune system as well as neuroendocrine signals (e.g., neuropeptides and stress hormones) can in turn shape the microbial community structure and activity, forming a closed-loop regulatory signaling network (19, 122). An in-depth study of the fine-tuned operation of this interplay network in different individuals will provide a key breakthrough in elucidating the pathogenesis of such diseases. Therefore, future studies will not only require the use of more powerful integrated multi-omics analyses to paint a panoramic picture, but advances in single-cell analysis technologies will also facilitate the acquisition of multidimensional information at the single-cell level (123, 124), precisely resolving the heterogeneity of different cell types and their specific roles in the microbiota-host interactions network (125). Based on the results of these insightful understandings, the ultimate goal is to develop novel diagnostic markers and design precise and personalized intervention strategies (e.g., probiotics or dietary therapies targeting microbial metabolism, immunomodulation, or gut-brain axis signaling, etc.) to target the dysfunctional mechanisms of the aforementioned interactions, opening new pathways for the prevention and treatment of FD.

### 6.3 Clinical translational prospects of FD and intestinal microecology research

Research on FD and intestinal microecology holds significant translational potential for clinical applications. First, investigations into gut microecology-derived biomarkers may enable early and precise diagnosis of FD. The identification of disease-associated microbial signatures, including specific gut microorganisms and their metabolic byproducts, could facilitate the development of rapid, accurate diagnostic tools (36, 49, 126). Such advancements would permit timely clinical intervention, potentially improving patient outcomes.

Second, dietary microbial modulation and microecological interventions represent promising therapeutic approaches for FD management. Empirical evidence demonstrates that targeted dietary fiber supplementation can elicit symptom-specific therapeutic effects through functional modulation of intestinal microbiota (127). Furthermore, microecological therapies, including probiotics and synbiotics, have exhibited clinical potential in FD treatment, likely mediated through modification of microbial metabolic functions (126, 128).

Emerging mechanistic insights into the FD-microbiota interaction are informing the development of next-generation probiotics, prebiotics, and personalized microecological treatment regimens. These innovations promise to deliver more precise, safer, and efficacious therapeutic options for FD patients. When integrated with novel diagnostic technologies—including artificial intelligence-assisted analysis and high-resolution imaging—these approaches enable comprehensive patient assessment and real-time treatment monitoring, thereby optimizing therapeutic outcomes.

Notably, findings from intestinal microecology research may have broader implications for managing other functional gastrointestinal disorders. This research trajectory may accelerate progress in clinical gastroenterology, potentially enhancing patient quality of life while alleviating the socioeconomic burden of gastrointestinal diseases.
